# Efficacy and safety of radiofrequency ablation for hyperparathyroidism: a meta-analysis and systematic review

**DOI:** 10.1007/s00330-025-11581-6

**Published:** 2025-04-17

**Authors:** So Yeong Jeong, Kyung Hoon Lee, Ji Ye Lee, Taehyuk Ham, Hunjong Lim, Minjung Ryu, Young Hun Jeon, Inpyeong Hwang, Tae Jin Yun, Jung Hee Kim, Se Jin Cho, Ji-hoon Kim

**Affiliations:** 1 Department of Radiology, Seoul National University Bundang Hospital, Seoul National University College of Medicine, Seongnam, Republic of Korea; 2 Department of Radiology, Kangbuk Samsung Hospital, Sungkyunkwan University School of Medicine, Seoul, Republic of Korea; 3 Department of Radiology, Seoul National University Hospital, Seoul National University College of Medicine, Seoul, Republic of Korea; 4 Department of Endocrinology and Metabolism, Seoul National University Hospital, Seoul National University College of Medicine, Seoul, Republic of Korea

**Keywords:** Radiofrequency ablation, Thermal ablation, Parathyroid lesion, Parathyroid gland, Hyperparathyroidism

## Abstract

**Objective:**

Radiofrequency ablation (RFA) is increasingly being investigated as a treatment for parathyroid lesions, with favorable outcomes, especially in patients who are ineligible for surgery or decline surgery. We aimed to assess the efficacy and safety of RFA in treating hyperparathyroidism.

**Materials and methods:**

PubMed and Embase were searched for original literature published on or before July 18, 2024. We included 14 eligible studies with 593 patients (241 with primary hyperparathyroidism [PHPT], 310 with secondary hyperparathyroidism [SHPT], and 42 with tertiary hyperparathyroidism [THPT]). Serial pooled means of biochemical indexes (parathyroid hormone [PTH], calcium, phosphorus), volume reduction ratio (VRR) after RFA, and complication rate were calculated.

**Results:**

In PHPT, the pooled mean baseline PTH value of 158.7 pg/mL and serum calcium value of 10.96 mg/dL significantly decreased to 57.3 pg/mL and 9.55 mg/dL, respectively, at 12 months (both *p* < 0.001), with both being within normal ranges. The pooled mean VRR gradually increased, reaching 95.6% at 12 months. In SHPT, the pooled mean baseline PTH value of 1683.7 pg/mL significantly decreased to 267.2 pg/mL at 12 months (*p* < 0.001), which was within the target reference level (PTH ≤ 585 pg/mL). In THPT, the mean baseline PTH value of 1284.9 pg/mL decreased to 161.6 pg/mL at 1 year (*p* < 0.001). The pooled incidence rates of total, major, and minor complications were 27.9%, 7.5%, and 20.0%, respectively.

**Conclusions:**

RFA showed promising effectiveness and safety profiles, particularly for patients who are ineligible for surgery or decline surgical intervention.

**Key Points:**

***Question*** *What is the efficacy and safety of RFA in treating hyperparathyroidism?*

***Findings*** *In PHPT, pooled mean values of biochemical indexes (serum PTH, calcium) were normal throughout 12-month follow-up. In SHPT and THPT, pooled mean PTH stayed within target ranges throughout 12-month follow-up.*

***Clinical relevance*** *RFA showed efficacy and safety in treating hyperparathyroidism, maintaining biochemical indexes within normal or target ranges throughout 12-month follow-ups. RFA would be a valuable treatment option for patients who are ineligible for surgery or who decline surgical intervention*.

**Graphical Abstract:**

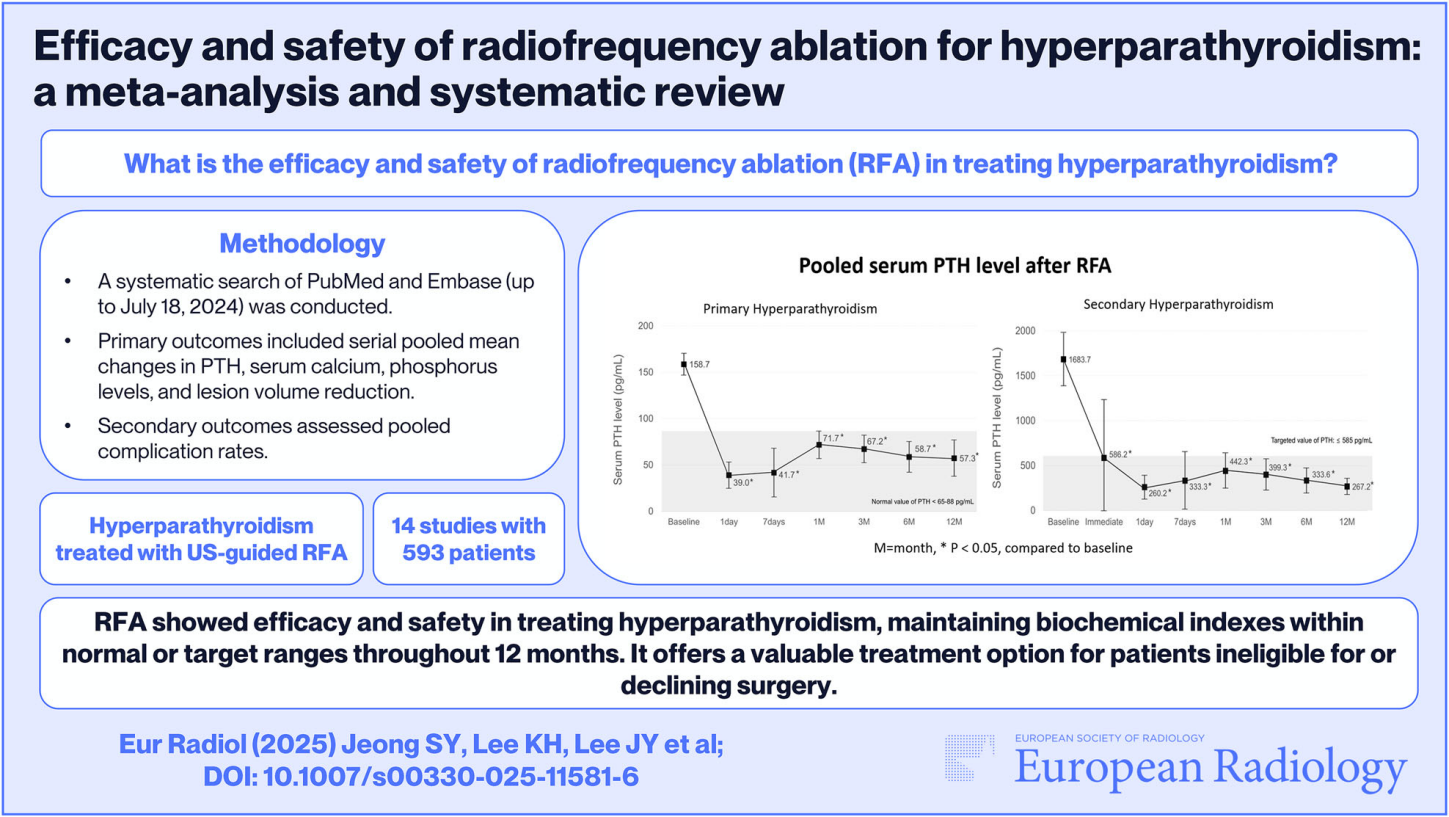

## Introduction

Hyperparathyroidism is a common endocrine disease characterized by elevated parathyroid hormone (PTH) levels, which disrupts normal calcium and phosphorus metabolism. It can be caused by either intrinsic changes involving excessive PTH excretion (primary hyperparathyroidism [PHPT] or tertiary hyperparathyroidism [THPT]) or extrinsic changes affecting calcium homeostasis (secondary hyperparathyroidism [SHPT]) [1]. Hyperparathyroidism results in various functional abnormalities, including nephrolithiasis, pathologic bone fractures, osteoporosis, peptic ulcer disease, pancreatitis, neuropsychiatric, and musculoskeletal symptoms, and thus requires treatment. Current guidelines recommend surgical intervention, specifically parathyroidectomy, as the first-line treatment course for patients with symptomatic PHPT and those with SHPT unresponsive to medical treatments [2, 3].

Although surgery is generally safe, patients with hyperparathyroidism often have serious comorbidities that increase the risk of postoperative complications and reduce tolerance to parathyroidectomy, especially among elderly patients [4, 5]. Given the successful outcomes and safety profiles of thermal ablation (TA) for malignant tumors of the lung, kidney, and liver [6–8], TA has been increasingly investigated as a treatment modality for parathyroid lesions in recent years. As an alternative to surgery, TA offers a minimally invasive treatment option, especially for patients who are ineligible for surgery or decline surgical intervention [9]. Among the various TA options, which include microwave ablation (MWA), radiofrequency ablation (RFA), laser ablation, and high-intensity focused ultrasound, MWA and RFA have been most frequently used for treating hyperparathyroidism.

Although there have been some recent studies that reported favorable outcomes following MWA or various TA methods for parathyroid lesions, studies specifically focusing on the efficacy and safety of RFA are notably absent. Therefore, this meta-analysis and systematic review aimed to consolidate and evaluate the published literature on the use of RFA for the treatment of hyperparathyroidism.

## Materials and methods

This meta-analysis and systematic review were performed according to the preferred reporting items for systematic reviews and meta-analyses (PRISMA) guidelines [10].

### Search methods and study selection

The PubMed/MEDLINE and EMBASE databases were systematically searched to identify published original literature reporting patients with parathyroid lesions who underwent treatment with RFA. The following search terms were used: ((hyperparathyroid*) OR (parathyroid) OR (hypercalcemia)) AND ((radiofrequency ablation) OR (radiofrequency therapy) OR (RFA)). The databases were searched for articles published on or before July 18, 2024.

The inclusion criteria were as follows: (1) patients with PHPT who underwent ultrasound (US)-guided RFA and had PTH levels higher than the upper normal limit, serum calcium levels higher than the upper normal limit, normal renal function tests, and parathyroid gland hyperplasia or adenoma diagnosed by US or radionuclide imaging; (2) patients with SHPT who underwent US-guided RFA; (3) patients with THPT who underwent US-guided RFA; (4) having detailed data sufficient for assessing pre-ablative and post-ablative follow-up clinical results including PTH, serum calcium, serum phosphorus, and volume of the index lesions; and (5) post-ablative follow-up results at least 6 months. The exclusion criteria were as follows: (1) case reports or case series including less than five patients; (2) conference abstracts, review articles, letters, editorials, comments, notes, short surveys, consensus statements, guidelines, or chapters; (3) confirmed or suspected overlapping of patient cohorts; and (4) non-English publications.

The search and study selection were conducted by two reviewers (S.Y.J. and K.H.L., with 6 years and 4 years of experience in head and neck imaging, respectively) with any disagreements being resolved through consensus.

### Data extraction

The following data were collected using standardized forms according to PRISMA guidelines [11]: (1) article characteristics: institution, country of origin, authors, year of publication, duration of patient recruitment, number of patients, age, male-to-female ratio, study design (prospective vs retrospective), follow-up schedule after ablation; (2) details regarding the ablation methods and RFA parameters: ablation electrode, ablation power, number of treatment sessions, ablation time; (3) details regarding clinical findings: pre-ablative and post-ablative volume of index lesions and laboratory results, including PTH, serum calcium, and serum phosphorus; and (4) details regarding complications. In the analysis, the measurement unit for serum PTH was pg/mL, and the measurement unit for serum calcium and phosphorus was mg/dL. Data were converted to these units as needed for consistency across studies. The normal reference values were < 65–88 pg/mL for PTH, 8.5–10.5 mg/dL for serum calcium, and 2.5–4.5 mg/dL for serum phosphorus. Complications were evaluated using the reporting standards of the Society of Interventional Radiology [12, 13]. Major complications were defined as adverse events associated with substantial morbidity or disability, an increased level of care, hospital admission, or substantial prolongation of hospital stay. All other complications were regarded as minor complications [12]. Two reviewers (S.Y.J. and K.H.L.) extracted and checked the data using a standardized form, with any disagreements being resolved through consensus.

### Quality assessment

Two reviewers independently performed quality assessment using the risk of bias for nonrandomized studies (RoBANS) tool [14, 15].

### Statistical methods

The primary outcome was the overall pooled mean and 95% confidence intervals (CIs) of PTH, serum calcium, and serum phosphorus levels (baseline, 1 day, 7 days, 1 month, 3 months, 6 months, and 12 months). Pooling was performed using the inverse variance method, with utilization of inverse variance weighting [16, 17]. Additionally, serial overall pooled volume reduction ratios (VRRs) of ablated index lesions for patients with PHPT were calculated (baseline, 1 month, 3 months, 6 months, and 12 months). *p* values were for comparisons between baseline and post-treatment values, with *p* < 0.05 being considered statistically significant. A secondary outcome was the pooled incidences of major and minor complications.

The *I*^2^ statistic was used to assess among-study heterogeneity. *I*^2^ > 50% and *p* < 0.10 were considered to indicate significant heterogeneity [18]. Meta-analyses with insignificant and significant heterogeneity were performed using the fixed- and random-effects models, respectively. All statistical analyses were conducted by one author (S.Y.J., with 4 years of experience in conducting meta-analyses) using the “meta” package in R version 4.4.1 (http://www.r-project.org/).

## Results

### Eligible studies and characteristics of included studies

**Fig. 1:**
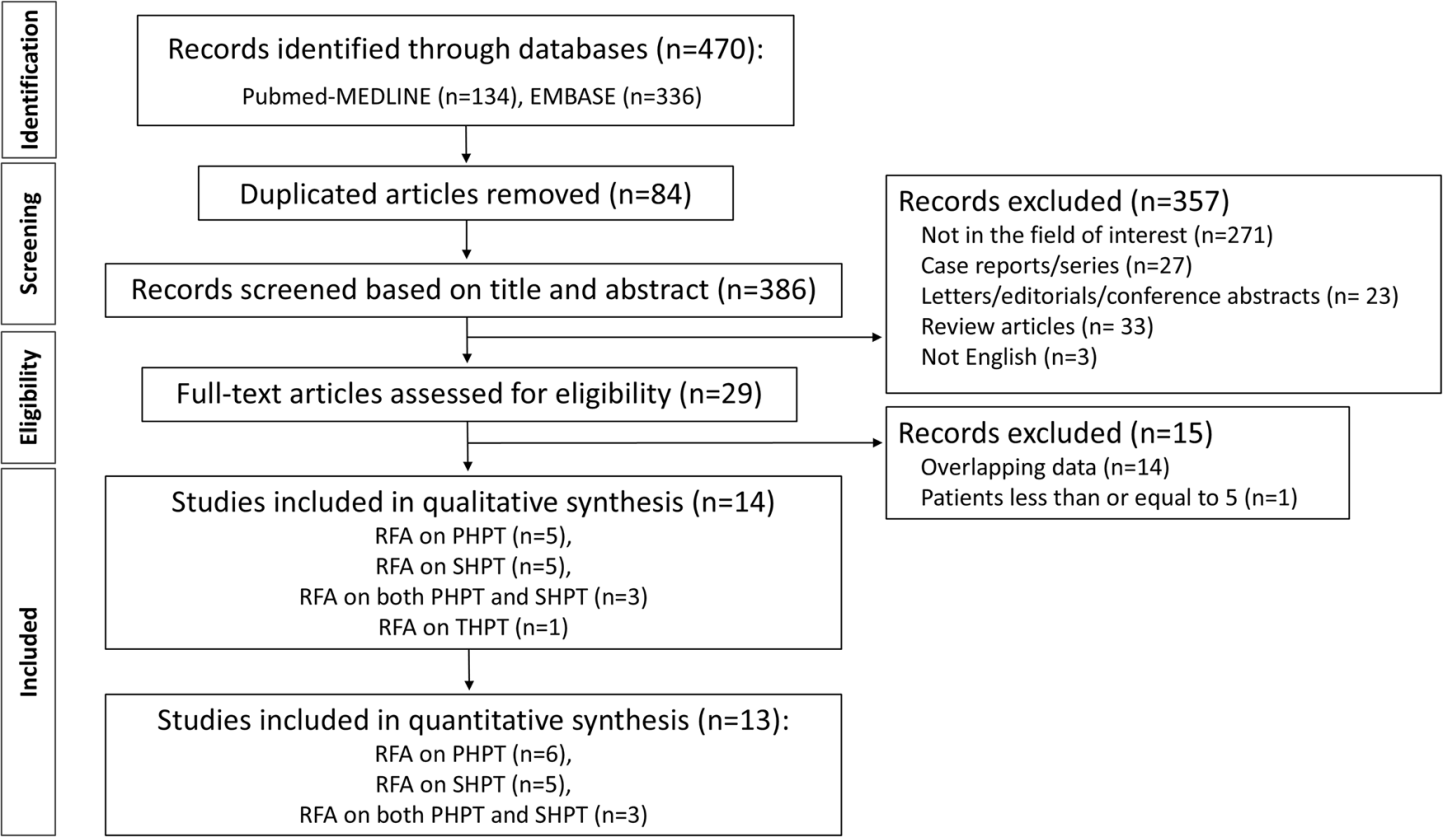
Flow diagram of the study selection process

The search of PubMed/MEDLINE and Embase resulted in the identification of 470 articles. After removing duplications, we further excluded 386 studies that did not meet the inclusion criteria were removed based on the title and abstract. After full-text screening of the remaining 29 studies, 15 studies were removed due to including fewer than five patients or overlapping cohorts with those of other studies. Finally, we included 14 eligible studies [9, 19–31] (Fig. 1).

Table 1 lists the characteristics of the 14 eligible studies, which included five studies on PHPT [21–24, 28], five on SHPT [19, 25–27, 29], three on both PHPT and SHPT [9, 20, 31], and one on THPT [30]. Four studies [9, 24, 29, 30] had multicenter designs, with the remaining studies being conducted in single centers. There were 13 retrospective studies and one [26] prospective study. The studies included 593 patients, including 241 patients with PHPT [9, 20–24, 28, 31], 310 patients with SHPT [9, 19, 20, 25–27, 29, 31], and 42 patients with THPT [30]. The number of patients in each study ranged from 7 to 165, with a mean age of 45.8–64.8 years. Table 1 describes the detailed follow-up schedules after RFA.

**Table 1:** Characteristics of the included studies

First author (year of publication)	Affiliation	No. of patients	Age (mean ± SD, year)	Male: female	Study design	No. of ablated lesions	Post-ablation follow-up schedule (h = hour, M = month)
PHPT							
Chehrehgosha [28]	Aja University of Medical Sciences, Iran	60	60.7 ± 12.6	13:47	Retro.	60	Baseline, 7 days, 1 M, 2 M, 3 M, 4 M, 6 M, and 8 M
Chen [31]	Kaohsiung Chang Gung Memorial Hospital, Taiwan	7	57.9 ± 12.6	2:5	Retro.	7	Baseline, 1 day, 7 days, 1 M, 3 M, 6 M, and 12 M
Ha [9]	Multicenter, Korea^a^	11	64.8 ± 15.0	2:9	Retro.	NA	Baseline, 1 M, 6 M, and 12 M
Khandelwal [21]	Medanta—the Medicity Hospital, India	10	55.2 ± 14.9	6:4	Retro.	10	PTH (baseline, 7 days, 6 M) Calcium (baseline, 72 h, 1 M, 6 M, 12 M, and 18 M)
Li [23]	Chinese People’s Liberation Army General Hospital, China	25	53.9 ± 10.9	3:22	Retro.	1 (21 pts) 2 (4 pts)	Baseline, 1 day, 1 M, 3 M, 6 M, and 12 M
Peng [24]	Multicenter, China^b^	51	58.0 ± 14.3	21:30	Retro.	51	Baseline, 1 day, 3 days, 1 M, 3 M, 6 M, and 12 M
León [22]	San Cecilio Clinical University Hospital of Granada, Spain	29	60.9 ± 13.3	7:22	Retro.	29	Baseline, 1 M, 3 M, 6 M, 12 M, and 24 M
Qiu [20]	The first Affiliated Hospital of Zhengzhou University, China	48	51.9 ± 14.0	8:40	Retro.	Single (47 pts) Multiple (1 pts)	Baseline, 2 h, 1 day, 1 M, 3 M, 6 M, and 12 M
SHPT							
Chen [31]	Kaohsiung Chang Gung Memorial Hospital, Taiwan	9	51.8 ± 16.0	6:3	Retro.	NA	Baseline, 1 day, 7 days, 1 M, 3 M, 6 M, and 12 M
Ha [9]	Multicenter, Korea^a^	8	61.6 ± 17.3	3:5	Retro.	1 (3 pts) 2 (2 pts) 3 (3 pts)	Baseline, 1 M, 6 M, and 12 M
Qin [26]	Nanchong Central Hospital, China	32	53.5 ± 13.6	13:19	Pro.	131	Baseline, immediate, 1 day, 2 days, 6 M, and 12 M
Ren [27]	Zhejiang Provincial People’s Hospital, China	47	51 ± 12	29:18	Retro.	173	Baseline, discharge date, 1 M, 3 M, 6 M, 12 M, and 24 M
Yue [29]	Multicenter, China^c^	165	Median 51	73.92	Retro.	582	Baseline, 1 M, 3 M, 6 M, 12 M, 36 M, and 60 M
Zhang [19]	Fujian Provincial Hospital, China	30	45.8 ± 13.3	16:14	Retro.	NA	Baseline, 1 day, 3 days, 1 M, 3 M, and 6 M
Qiu [20]	The first Affiliated Hospital of Zhengzhou University, China	9	56.4 ± 17.0	5:4	Retro.	Single (5 pts) Multiple (4 pts)	Baseline, 2 h, 1 day, 1 M, 3 M, 6 M, and 12 M
Jiang [25]	Shanghai Tenth People’s Hospital, China	10	51 ± 8.5	1:9	Retro.	20	Baseline, 7 days, 6 M, 12 M, and 36 M
TPHT							
Deng [30]	Multicenter, China^d^	42	Median 54.5	17:21	Retro.	1 (1 pts) 2 (4 pts) 3 (6 pts) 4 (26 pts) 5 (1 pts)	Baseline, 1 day, 1 M, 3 M, 6 M, 12 M, 24 M, 36 M, and 48 M

*NA* not available, *PHPT* primary hyperparathyroidism, *Retro.* retrospective, *Pro.* prospective, *PTH* parathyroid hormone, *SHPT* secondary hyperparathyroidism, *THPT* tertiary hyperparathyroidism

^a^ Asan Medical Center, Haeundae Sharing and Happiness Hospital, Ajou University Medical Center, Korea

^b^ Hangzhou Hospital of Traditional Chinese Medicine, Zhejiang Provincial People’s Hospital, China

^c^ Shanghai Tenth People’s Hospital, Zhongshan Hospital, Zhejiang Provincial People’s Hospital, Hangzhou Hospital of Traditional Chinese Medicine, The First Affiliated Hospital of Anhui Medical University, Yiwu Fuyuan Hospital, The First People’s Hospital of Aksu, China

^d^ Shanghai Tenth People’s Hospital, Hangzhou Hospital of Traditional Chinese Medicine, and Zhejiang Provincial People’s Hospital, China

### Characteristics of the ablation methods

Table 2 presents the characteristics of the ablation methods. In all studies, the RFA procedures were performed under local anesthesia without sedation, with the hydrodissection technique being commonly applied. Active tips of various sizes, including 3.8 mm, 5 mm, 7 mm, or 9 mm, were used. The mean ablation power ranged from 10 W to 70 W, with single or multiple (two or three) treatment sessions.

**Table 2:** Characteristics of the ablation methods

First author (year of publication)	Ablation electrode	Ablation energy [power range]	Treatment session	Ablation time (min.)
PHPT				
Chehrehgosha [28]	18 G: 5 mm, 7 mm active tip	NA [25–75 W]	NA	NA
Khandelwal [21]	17 G: 5 mm active tip	NA [40–80 W]	NA	NA
Li [23]	18 G: 9 mm active tip	5.67 ± 1.58Kcal [NA]	One (*n* = 22), two (*n* = 3)	3.9 ± 2.9
Peng [24]	18 G: 7 mm active tip	NA [30–45 W]	NA	NA
León [22]	NA	NA [10–70 W]	One (NA), two (NA)	NA
SHPT				
Yue [29]	18 G: 7 mm active tip	35 W	One (*n* = 116), two (*n* = 49)	NA
Qin [26]	18 G: 7 mm active tip	NA	One (*n* = 32)	NA
Ren [27]	18 G: 7 mm active tip	NA	One (*n* = 26), two (*n* = 21)	NA
Zhang [19]	18 G: 7 mm active tip	NA [set at 35 W]	NA	NA
Jiang [25]	18 G: 7 mm active tip	NA [30–35 W]	One (*n* = 9), two (*n* = 1)	NA
PHPT and SHPT				
Chen [31]	18 G: 5 mm or 7 mm active tip	2.98 ± 3.25Kcal [NA]	One (*n* = 9)	14.30 ± 8.3
Ha [9]	18 or 19 G: 3.8, 5, 7, or 10 mm active tip	NA [10–70 W]	1.6 ± 0.9 (1–3 sessions per nodule)	3.3 ± 1.3
Qiu [20]	18 G: 5 mm active tip	NA [20–40 W]	NA	NA
THPT				
Deng [30]	18 G: 7 mm active tip	35 W	One (*n* = 25) two (*n* = 13)	2.9 ± 1.0

*NA* not available, *PHPT* primary hyperparathyroidism, *SHPT* secondary hyperparathyroidism, *THPT* tertiary hyperparathyroidism

### Outcomes of ablation in PHPT

**Fig. 2:**
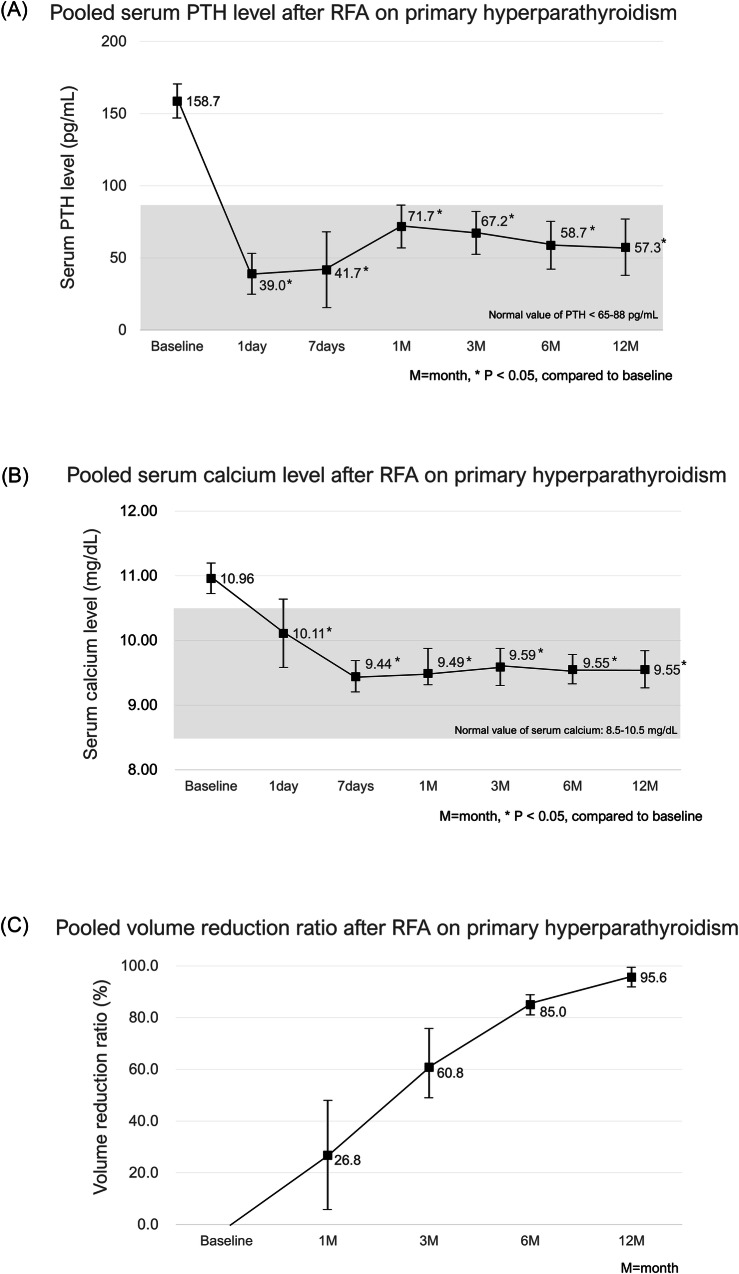
Pooled laboratory results of baseline and respective follow-up results of PTH (**A**) and calcium (**B**) levels, and pooled results of VRR (**C**) in PHPT

Figure 2(A, B) and Supplementary Table 1 present the pooled laboratory results of baseline and follow-up data regarding PTH and serum calcium levels in patients with PHPT. *p* values were calculated in comparison with baseline values. All included studies reported data regarding PTH and serum calcium levels. There were insignificant heterogeneities among the baseline data regarding PTH levels and 7-day follow-up data regarding serum calcium levels (*p* = 0.35, *I*^2^ = 9.8%, and *p* = 0.90, *I*^2^ = 0%, respectively); therefore, the fixed-effects models were used. However, there were significant heterogeneities among other data (*p* < 0.05, *I*^2^ > 50%), and thus the random-effects models were applied.

PTH levels showed a significant decrease from baseline (158.7 pg/mL, 95% CI: 146.8–170.5) throughout the 12-month follow-up period after RFA (all, *p* < 0.001). There was a marked initial reduction, with the PTH level dropping to 39.0 pg/mL (95% CI: 25.0–53.0 pg/mL) within 1 day and 41.7 pg/mL (95% CI: 15.5–67.9 pg/mL) within 7 days after RFA. There was a slight increase in the PTH level at 1 month after RFA (71.7 pg/mL, 95% CI: 56.9–86.5); however, it was within the normal reference range (< 65–88 pg/mL). However, PTH levels subsequently stabilized and remained within the normal range, with mean levels of 67.2 pg/mL (95% CI: 52.4–82.1) at 3 months, 58.7 pg/mL (95% CI: 42.1–75.3) at 6 months, and 57.3 pg/mL (95% CI: 37.9–76.6) at 12 months after RFA.

The mean serum calcium levels showed a significant decrease from baseline (10.96 mg/dL, 95% CI: 10.72–11.20) throughout the 12-month follow-up period after RFA (all, *p* < 0.001, except *p* = 0.004 at 1 month). There was a marked initial reduction, with the serum calcium level dropping to 10.11 mg/dL (95% CI: 9.58–10.64) within 1 day and 9.44 mg/dL (95% CI: 9.20–9.68) within 7 days after RFA, which are both within the normal reference range (8.5–10.5 mg/dL). Serum calcium levels remained stable at subsequent follow-ups, with values of 9.49 mg/dL (95% CI: 9.31–9.88) at 1 month, 9.59 mg/dL (95% CI: 9.30–9.88) at 3 months, 9.55 mg/dL (95% CI: 9.33–9.78) at 6 months, and 9.55 mg/dL (95% CI: 9.26–9.84) at 12 months after RFA.

Five studies reported the serial mean VRR with standard deviation values after RFA [20, 23, 24, 28, 31]. Figure 2(C) presents the pooled results of the respective follow-up VRR values. The pooled mean VRR gradually increased and reached 95.6% (95% CI: 91.8–99.5) at 12 months.

### Outcomes of ablation in SHPT

**Fig. 3:**
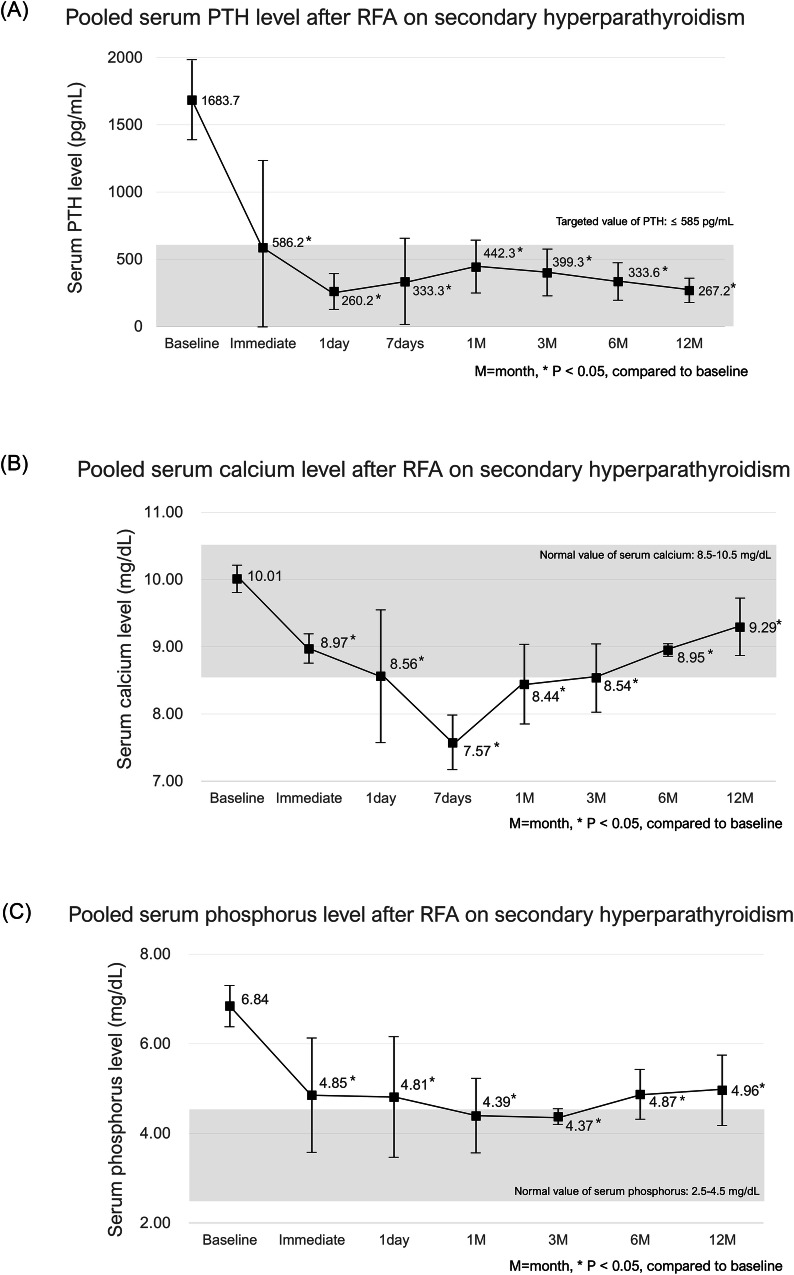
Pooled laboratory results of baseline and respective follow-up results of PTH (**A**), calcium (**B**), and phosphorus (**C**) levels in SHPT

Figure 3(A–C) and Supplementary Table 2 present the pooled laboratory results of baseline and respective follow-up results of PTH, serum calcium, and serum phosphorus levels in patients with SHPT. All included studies reported data regarding PTH levels. In patients with SHPT, the targeted PTH reference value was ≤ 585 pg/mL (no more than nine times the upper limit of normal was chosen in agreement with Kidney Disease: Improving Global Outcomes guidelines [32]). There were significant among-study heterogeneities in the pooled results at all respective follow-ups (*p* < 0.05, *I*^2^ > 50%); therefore, random-effects models were used.

The mean PTH levels showed a significant decrease from baseline (1683.7 pg/mL, 95% CI: 1387.6–1979.7) throughout the 12-month follow-up period after RFA (all, *p* < 0.001). There was a marked initial reduction, with the PTH level dropping to 586.2 pg/mL (95% CI: −60.2 to 1232.7) immediately after RFA. Further decreases were observed within 1 day (260.2 pg/mL, 95% CI: 126.4–394.1) and at 7 days (333.3 pg/mL, 95% CI: 12.5–654.0) after RFA. There was a slight increase in PTH levels at 1 month (442.3 pg/mL, 95% CI: 246.3–638.3) and 3 months (399.3 pg/mL, 95% CI: 225.0–573.6) after RFA. However, PTH levels subsequently stabilized, with values of 333.6 pg/mL (95% CI: 193.2–474.0) at 6 months and 267.2 pg/mL (95% CI: 178.0–356.4) at 12 months after RFA.

All included studies reported data regarding serum calcium levels. There were no significant among-study heterogeneities in the immediate, 7-day, and 6-month follow-up data (*p* = 0.44, *I*^2^ = 0%, *p* = 0.76, *I*^2^ = 0%, and *p* = 0.41, *I*^2^ = 2.3%, respectively), and thus fixed-effects models were applied. However, there were significant among-study heterogeneities in the other data (*p* < 0.05, *I*^2^ > 50%); therefore, we used the random-effects models.

The mean serum calcium levels showed a significant decrease from baseline (10.01 mg/dL, 95% CI: 9.80–10.21) throughout the 12-month follow-up period after RFA (all *p* < 0.001, except *p* = 0.004 at 12 months). There was a marked initial reduction, with serum calcium levels dropping to 8.97 mg/dL (95% CI: 8.75–9.19) immediately after RFA and further decreasing to 7.57 mg/dL (95% CI: 7.17–7.98) at 7 days after RFA, which was below the normal range (8.5–10.5 mg/dL). Subsequently, serum calcium levels gradually returned to the normal range, with values of 8.44 mg/dL (95% CI: 7.85–9.03) at 1 month, 8.54 mg/dL (95% CI: 8.03–9.04) at 3 months, 8.95 mg/dL (95% CI: 8.86–9.04) at 6 months, and 9.29 mg/dL (95% CI: 8.87–9.72) at 12 months after RFA.

Four studies reported data regarding serum phosphorus [19, 26, 27, 29]. There were significant among-study heterogeneities in all values at all respective follow-ups (*p* < 0.05, *I*^2^ > 50%), and thus the random-effects model was used.

The mean serum phosphorus levels showed a significant decrease from baseline (6.84 mg/dL, 95% CI: 6.38–7.30) throughout the 12-month follow-up period after RFA (*p* < 0.001, except for *p* = 0.005 at 1 month). There was a marked initial reduction, with phosphorus levels dropping to 4.85 mg/dL (95% CI: 3.57–6.12) immediately after RFA and 4.81 mg/dL (95% CI: 3.46–6.16) within 1 day. Moreover, they further decreased to 4.39 mg/dL (95% CI: 3.56–5.23) at 1-month and 4.37 mg/dL (95% CI: 4.19–4.55) at 3-month follow-ups, which were within the normal range (2.5–4.5 mg/dL). Subsequently, serum phosphorus levels gradually increased above the normal range, with values of 4.87 mg/dL (95% CI: 4.32–5.42) at 6 months and 4.96 mg/dL (95% CI: 4.17–5.75) at 12 months after RFA.

### Outcomes of ablation in THPT

One study included 36 patients with THPT [30]. Among them, the mean value of baseline intact PTH was 1284.9 pg/mL (95% CI: 996.5–1896.2), which decreased to 161.6 pg/mL (95% CI: 118.0–435.5) at 1 year after RFA (*p* < 0.001). The mean baseline serum calcium level was 10.58 mg/dL (95% CI: 10.34–10.9), which decreased to 8.9 mg/dL (95% CI: 8.4–9.38) at 1 year after RFA (*p* < 0.001). The mean baseline serum phosphorus level was 6.91 mg/dL (95% CI: 5.36–8.14), which decreased to 4.74 mg/dL (95% CI: 4.09–5.17) at 1 year after RFA (*p* < 0.001).

### Therapeutic success rate

Among the 14 studies, 10 reported therapeutic success with varying criteria [9, 21–24, 26, 27, 29–31] (Table 3). For patients with PHPT, six studies reported therapeutic success rates from 48.3% to 100% [9, 19, 21–24]. Chen et al [31] reported a 100% success rate at 1 year, based on significant volume reduction and biochemical remission. Ha et al [9] used therapeutic success criteria of residual volume < 0.1 mL and chemical remission, reporting a 63.6% therapeutic success rate at 1 year. Li et al [23] and León et al [22] reported 84% and 48.3% therapeutic success rates at 1 year and 2 years, respectively, using criteria of normalization of PTH and calcium levels. Khandelwal et al [21] and Peng et al [24] reported 60% and 98% therapeutic success rates at 18 months and 1 year, respectively, using the criterion of normalization of calcium levels. Among patients with SHPT, five studies reported therapeutic success rates ranging from 37.5% to 78.2% [9, 19, 20, 26, 29]. Three studies defined therapeutic success as achieving a PTH level below 300 pg/mL, with therapeutic success rates of 37.5% at 1 year, 57% at 1 year, and 64.1% at 2 years [9, 19, 27]. Qin et al [26] reported a 71.9% therapeutic success rate at 1 year based on complete ablation on contrast-enhanced US and PTH level below 300 pg/mL. Yue et al [29] reported a 78.2% therapeutic success rate at 51 months based on PTH level below 585 pg/mL. In the study on patients with THPT, Deng et al [30] reported an 86.4% therapeutic success rate at 1 year based on achieving a PTH level below 585 pg/mL.

**Table 3:** Reported therapeutic success rate in the included studies

First author (year of publication)	Therapeutic success criteria	Success rate	Follow-up period
PHPT			
Chen [31]	Significant volume reduction and biochemical remission	100%	1 year
Ha [9]	Residual volume < 0.1 mL and chemical remission	63.6%	1 year
Li [23]	Normalization of PTH and calcium levels	84%	1 year
León [22]	Normalization of PTH and calcium levels	48.3%	2 years
Khandelwal [21]	Normalization of calcium levels	60%	18 months
Peng [24]	Normalization of calcium levels	98%	1 year
SHPT			
Chen [19]	PTH < 300 pg/mL	57%	1 year
Ha [9]	PTH < 300 pg/mL	37.5%	1 year
Ren [27]	PTH < 300 pg/mL	64.1%	2 years
Yue [29]	PTH < 585 pg/mL	78.2%	51 months
Qin [26]	Complete ablation on contrast-enhanced US and PTH < 300 pg/mL	71.9%	1 year
THPT			
Deng [30]	PTH < 585 pg/mL	86.4%	1 year

*PHPT* primary hyperparathyroidism, *PTH* parathyroid hormone, *SHPT* secondary hyperparathyroidism, *THPT* tertiary hyperparathyroidism

### Safety

All included studies reported RFA-related complications. Table 4 describes details regarding the complications. The reported major complications included permanent voice change, severe or permanent hypocalcemia, and permanent hypoparathyroidism. The minor complications included transient voice change, mild or transient hypocalcemia, transient hypoparathyroidism, hematoma, and infections. We evaluated the pooled incidence of complications in 13 studies with 444 patients that reported an incidence of complications per patient [9, 19–28, 30, 31]. The overall pooled incidences of total, major, and minor complications were 27.9% (95% CI: 14.1–47.6%), 7.5% (95% CI: 3.6–15.1%), and 20.0% (95% CI: 10.2–35.5%), respectively. Heterogeneity was observed in the total (*I*^2^ = 89.4%), major (*I*^2^ = 60.4%), and minor (*I*^2^ = 88.0%) complications data. In the subgroup analysis, the incidences of total and major complications were significantly higher in patients with SHPH than in patients with PHPH (total complications; 13.0% [(95% CI: 4.7–30.9%] vs 45.0% [(95% CI: 25.2–66.5%], *p* = 0.18 and major complications; 2.7% [(95% CI: 1.1–6.7%] vs 21.1% [(95% CI: 15.0–28.8%], *p* < 0.001). The incidence of minor complications was higher in patients with SHPH than in patients with PHPH, but there was no statistical significance (12.1% [(95% CI: 4.2–30.0%] vs 28.5% [(95% CI: 14.2–49.1%], *p* = 0.145).

**Table 4:** Reported complications in the included studies

First author (year of publication)	No. of patients	No. of complications	No. of major complications	No. of minor complications
PHPT				
Chehrehgosha [28]	60	3	0	2 Hematoma 1 Transient voice change
Chen [31]	7	1	1 Permanent voice change	0
Ha [9]	11	1	0	1 Transient hypocalcemia
Khandelwal [21]	10	1	0	1 Transient voice change
Li [23]	25	1	0	1 Transient hypocalcemia
Peng [24]	51	38	0	3 Transient voice change 8 Transient hypocalcemia 27 Transient hypoparathyroidism
León [22]	29	2	0	2 Transient voice change
Qiu [20]	48	5	0	3 Transient voice change 2 Transient hypocalcemia
SHPT				
Chen [31]	9	2	2 Permanent voice change	0
Ha [9]	8	2	1 Permanent voice change	1 Transient hypocalcemia
Yue [29]	165 (214 sessions)		37 Sessions severe hypocalcemia 1 Session permanent hypoparathyroidism	3 Sessions hematoma 16 Sessions transient voice change 61 Sessions transient hypocalcemia
Qin [26]	32	9	0	9 Transient voice change
Ren [27]	47	42	6 Permanent voice change 6 Severe hypocalcemia	1 Hematoma 3 Fever, infection 26 Transient hypocalcemia
Zhang [19]	30	14	6 Severe hypocalcemia	8 Transient hypocalcemia
Qiu [20]	9	5	0	2 Transient voice change 1 Hematoma 2 Transient hypocalcemia
Jiang [25]	30	10	2 Permanent voice change 1 Permanent hypoparathyroidism 4 Severe hypocalcemia	3 Transient hypocalcemia
THPT				
Deng [30]	38		4 Permanent hypocalcemia	1 Hematoma 3 Transient voice change 13 Transient hypocalcemia 1 Transient hypoparathyroidism

*PHPT* primary hyperparathyroidism, *SHPT* secondary hyperparathyroidism, *THPT* tertiary hyperparathyroidism

### Assessment of the study quality

**Fig. 4:**
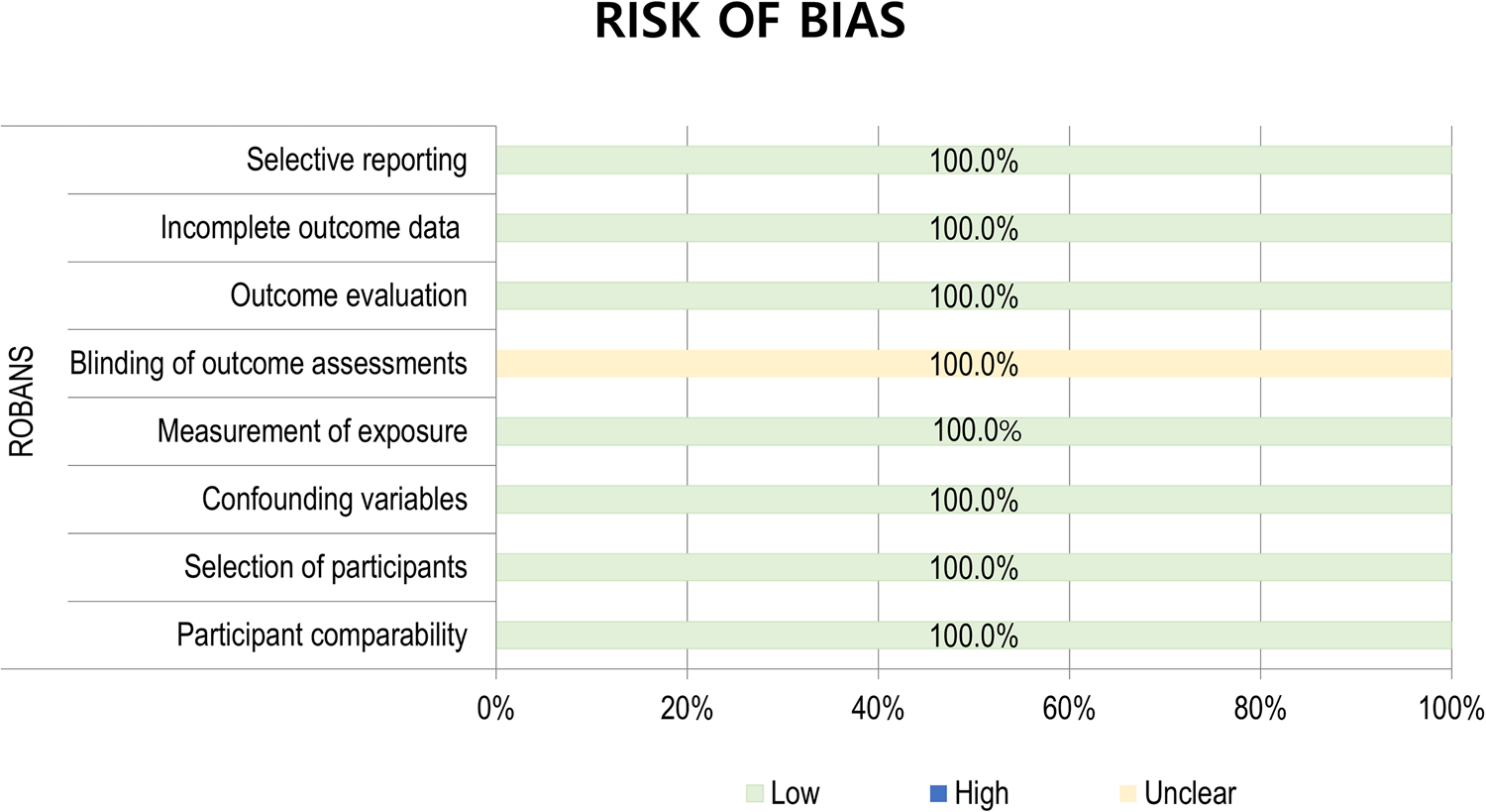
Quality assessment of the included studies according to the RoBANS

In quality assessment according to RoBANS criteria (Fig. 4), all 14 studies had a low risk of bias in the selective reporting, incomplete outcome data, outcome data, measurement of exposure, confounding variables, selection of participants, and participant comparability domains. However, all studies showed an unclear risk of bias in blinding of the outcome assessment domain since they did not make clear statements regarding patient/investigator blinding.

## Discussion

This meta-analysis uniquely evaluated the efficacy and safety of RFA among various TA techniques across the complete spectrum of hyperparathyroidism, encompassing primary, secondary, and tertiary subtypes. The results demonstrated a significant efficacy of RFA in treating hyperparathyroidism. In patients with PHPT, the mean pooled PTH and serum calcium levels were effectively controlled after RFA, reaching the normal range and remaining for 12 months. In patients with SHPT, RFA significantly decreased PTH and phosphorus levels, as well as normalized serum calcium levels for 12 months. As for therapeutic success rate, RFA resulted in 42.3–100% and 37.5–78.2% in patients with PHPT and SHPT, respectively. The overall pooled incidences of total, major, and minor complications were 27.9%, 7.5%, and 20.0%, respectively.

Currently, parathyroidectomy is the definitive treatment for PHPT and is recommended for medically intractable SHPT or THPT by guidelines. However, RFA offers numerous advantages such as local anesthesia, reduced blood loss, shortened procedure time, no scarring, shortened hospitalization duration, reduced risk of postprocedural infections, ability to undergo repeated procedures, and reduced postprocedural pain [27, 33, 34]. These benefits make RFA an excellent option for patients who are ineligible for surgery due to comorbidity. RFA can achieve efficacy almost equivalent to surgery in such cases, representing a decisive advantage. Two studies comparing MWA and parathyroidectomy in patients with PHPT reported comparable therapeutic success and complication rates [34, 35]. In patients with SHPT, several studies comparing TA such as MWA and RFA with parathyroidectomy have demonstrated that they are both effective and safe [27, 33, 34, 36–38]. However, TA generally presents a higher risk of SHPT persistence or recurrence but a lower risk of hypocalcemia than parathyroidectomy [38].

Our findings showed that RFA was more effective and involved significantly lower complication rates in patients with PHPT than in patients with SHPT. This can be primarily attributed to PHPT, typically involving a single parathyroid adenoma, while SHPT often requires treatment of all four parathyroid glands. Therefore, SHPT is expected to have a higher risk of complications since it requires treatment for more nodules and more treatment sessions, and these patients typically have poor underlying medical conditions such as chronic kidney disease. In this context, RFA could be more effectively applied in cases of recurrent SHPT after surgery [39].

Regarding THPT, there is limited available literature regarding the use of TA, with only one and two studies on RFA and MWA, respectively [29, 40, 41]. However, all these studies reported favorable outcomes. Given the similarities between SHPT and THPT in terms of gland multiplicity, it is reasonable to expect comparable efficacy and safety profiles for TA in patients with THPT. This is particularly relevant since both patients with THPT and SHPT often face challenges with surgical interventions attributable to their medical conditions. Although the rarity of THPT inherently limits the available evidence, it is important to consider the therapeutic potential of TA.

In our study, we analyzed the serial changes of pooled biochemical indexes after RFA. In patients with PHPT, PTH levels immediately decreased after RFA, while serum calcium levels showed a more gradual decrease. Even after normalization of serum calcium levels, there was a tendency for PTH levels to slightly elevate within 1–3 months, which may be attributed to a rebound effect [24]. However, both PTH and serum calcium levels were consistently maintained within normal reference levels throughout the 12 follow-up months. In patients with SHPT, the immediate decrease in PTH and serum calcium levels led to hypocalcemic status at 7 days after RFA. Subsequently, similar to PHPT, PTH levels showed a slight elevation between 1–3 months, but remained within the target reference range. Meanwhile, serum calcium levels returned to normal range at the 6-month and 12-month follow-up. This rebound effect could be explained by the temporary imbalance between bone catabolism and anabolism after rapid PTH decline, leading to compensatory PTH secretion from residual parathyroid tissue [24]. Understanding these general laboratory changes is crucial for the prediction of prognosis, the evaluation of efficacy, and the assessment of the need for calcium and vitamin D supplements.

Although not as frequent as in surgery, hoarseness and hypocalcemia remain significant concerns during and immediately after RFA [27, 33–38]. The size, number, and location of the parathyroid lesions, in addition to the ablation time, power, and machine, might influence the development of hoarseness [42]. As for hypocalcemia, high alkaline phosphatase was reported to be an important risk factor, especially in SHPT [27]. To prevent complications from damage to the surrounding major structures during the procedure, it is essential to actively use the hydrodissection technique (injection of a 5% dextrose solution between the nerve and target lesion) and ensure that the practitioner is well-acquainted with neck anatomy. Additionally, to mitigate the risk of postoperative hypocalcemia, preoperative vitamin D supplementation to normal levels can enhance the compensatory capacity in order to maintain calcium homeostasis following sudden decreases in PTH levels after RFA.

The favorable outcomes of RFA for hyperparathyroidism are comparable to those of MWA [40, 43]. Although both MWA and RFA are TA techniques, MWA offers potentially greater power and effect but may involve higher complication risks, whereas RFA provides more precise control with potentially lower risks. Despite these differences, previous studies observed no significant between-method differences in the effectiveness or complication rates [44–46]. However, RFA may be more advantageous than MWA in treating small parathyroid lesions near critical structures such as the recurrent laryngeal nerve. The more precise and delicate control offered by RFA may make it a safer and more suitable option for these procedures, especially for less experienced operators.

This study has several limitations. First, although we included all available studies at the time of data collection, a small number of eligible studies were included. Second, most of the data showed heterogeneity. Third, the follow-up duration was relatively short. A long-term follow-up study is required to determine the long-term effectiveness of RFA treatment for hyperparathyroidism. However, we excluded studies with results of less than six months and included studies with follow-up results of six months or longer, aiming to analyze longer-term outcomes compared to previous meta-analyses. Fourth, only cohort-level data were available for the meta-analyses, with patient-level data being unavailable, which limited further evaluation of the overall therapeutic success rate and made it challenging to perform robust subgroup analyses. Further well-designed studies are warranted to solidify the role of RFA in managing patients with hyperparathyroidism across PHPT, SHPT, and THPT, especially for patients unsuitable for surgery.

In conclusion, based on the available evidence, RFA showed promising effectiveness and safety profiles, particularly for patients with hyperparathyroidism who are ineligible for surgery or decline surgical intervention.

## Supplementary information

ELECTRONIC SUPPLEMENTARY MATERIAL

## Abbreviations

CI: Confidence interval
MWA: Microwave ablation
PHPT: Primary hyperparathyroidism
PTH: Parathyroid hormone
RFA: Radiofrequency ablation
SHPT: Secondary hyperparathyroidism
TA: Thermal ablation
THPT: Tertiary hyperparathyroidism
VRR: Volume reduction ratio
