# Supplementary material for: Efficacy and safety of radiofrequency ablation for hyperparathyroidism: a meta-analysis and systematic review
Source: Eur Radiol. 2025 Apr 17;35(10):6583–97. doi: 10.1007/s00330-025-11581-6 (PMC12417237; doi:10.1007/s00330-025-11581-6)
Supplement: Supplementary file 1 — ELECTRONIC SUPPLEMENTARY MATERIAL [file 330_2025_11581_MOESM1_ESM.pdf]

Efficacy and Safety of Radiofrequency Ablation for Hyperparathyroidism: A Meta-Analysis and Systematic Review

ELECTRONIC SUPPLEMENTARY MATERIAL

Supplementary Table S1. Pooled laboratory results of baseline and respective follow-up results of parathyroid hormone and serum calcium levels and pooled results of volume reduction ratio in primary hyperparathyroidism.

	**PTH (pg/mL)**				**Serum calcium (mg/dL)**				**Volume reduction ratio (%)**		
	No. of studies	Pooled estimate [95% CI]	I^2	*P*	No. of studies	Pooled estimate [95% CI]	I^2	*P*	No. of studies	Pooled estimate [95% CI]	I^2
**Baseline**	8	158.7 [146.8–170.5]	9.8		8	10.96 [10.72–11.20]	68.4				
**1 day**	6	39.0 [25.0–53.0]	94.5	< 0.0001	6	10.11 [9.58–10.64]	93.2	0.0043			
**7 days**	2	41.7 [15.5–67.9]	96.6	< 0.0001	2	9.44 [9.20–9.68]	0	< 0.0001			
**1 month**	7	71.7 [56.9–86.5]	80.3	< 0.0001	8	9.49 [9.31–9.88]	78.7	< 0.0001	5	26.8 [5.7–47.9]	97.1
**3 months**	6	67.2 [52.4–82.1]	89.5	< 0.0001	6	9.59 [9.30–9.88]	82.8	< 0.0001	5	60.8 [48.9–72.6]	91.9
**6 months**	8	58.7 [42.1–75.3]	95.3	< 0.0001	8	9.55 [9.33–9.78]	87.0	< 0.0001	5	85.0 [81.0–88.9]	45.7
**12 months**	6	57.3 [37.9–76.6]	89.1	< 0.0001	7	9.55[9.26–9.84]	91.6	< 0.0001	4	95.6 [91.8–99.5]	63.7

P values are in comparison with baseline. PTH = parathyroid hormone

Supplementary Table S2. Pooled laboratory results of baseline and respective follow-up results of parathyroid hormone, serum calcium, and serum phosphorus levels in secondary hyperparathyroidism.

	PTH (pg/mL)				Serum calcium (mg/dL)				Serum phosphorus (mg/dL)			
	No. of studies	Pooled estimate [95% CI]	I^2	*P*	No. of studies	Pooled estimate [95% CI]	I^2	*P*	No. of studies	Pooled estimate [95% CI]	I^2	*P*
**Baseline**	7	1683.7 [1387.6–1979.7]	81.8		8	10.01[9.80–10.21]	57.0		4	6.84 [6.38–7.30]	70.1	
**Immediate**	3	586.2 [-60.2–1232.7]	81.1	0.0025	3	8.97 [8.75–9.19]	0	< 0.0001	2	4.85 [3.57–6.12]	95.5	0.0039
**1 day**	5	260.2 [126.4–394.1]	89.0	< 0.0001	6	8.56 [7.57–9.55]	95.3	0.0049	3	4.81 [3.46–6.16]	95.0	0.0053
**7 days**	2	333.3 [12.5–654.0]	87.7	< 0.0001	3	7.57 [7.17–7.98]	0	< 0.0001				
**1 month**	6	442.3 [246.3–638.3]	84.3	< 0.0001	6	8.44 [7.85–9.03]	77.3	< 0.0001	3	4.39 [3.56–5.23]	93.2	< 0.0001
**3 months**	5	399.3 [193.2–474.0]	79.5	< 0.0001	5	8.54 [8.03–9.04]	76.4	< 0.0001	3	4.37 [4.19–4.55]	0	< 0.0001
**6 months**	7	333.6 [193.2–474.0]	91.2	< 0.0001	8	8.95 [8.86–9.04]	2.3	< 0.0001	4	4.87 [4.32–5.42]	83.3	< 0.0001
**12 months**	6	267.2 [178.0–356.4]	88.3	< 0.0001	6	9.29 [8.87–9.72]	88.8	0.0032	3	4.96 [4.17–5.75]	93.0	< 0.0001

P values are in comparison with baseline. PTH = parathyroid hormone
